# Image Generation for 2D-CNN Using Time-Series Signal Features from Foot Gesture Applied to Select Cobot Operating Mode

**DOI:** 10.3390/s21175743

**Published:** 2021-08-26

**Authors:** Fadwa El Aswad, Gilde Vanel Tchane Djogdom, Martin J.-D. Otis, Johannes C. Ayena, Ramy Meziane

**Affiliations:** 1 Laboratory of Automation and Robotic interaction (LAR.i), Department of Applied Sciences, Université du Québec à Chicoutimi (UQAC), 555 Boulevard de l’Université, Chicoutimi, QC G7H 2B1, Canada; fadwa.lasswed@gmail.com (F.E.A.); gilde-vanel.tchane-djogdom1@uqac.ca (G.V.T.D.); Ramy1_Meziane@uqac.ca (R.M.); 2 Technological Institute of Industrial Maintenance (ITMI), Sept-Iles College, 175 Rue de la Vérendrye, Sept-Iles, QC G4R 5B7, Canada; 3 Communications and Microelectronic Integration Laboratory (LACIME), Department of Electrical Engineering, École de Technologie Supérieure, 1100 Rue Notre-Dame Ouest, Montréal, QC H3C1K3, Canada; cossoun-johannes.ayena.1@ens.etsmtl.ca

**Keywords:** human–robot collaboration, instrumented insole, foot gesture recognition, convolutional neural network

## Abstract

Advances in robotics are part of reducing the burden associated with manufacturing tasks in workers. For example, the cobot could be used as a “third-arm” during the assembling task. Thus, the necessity of designing new intuitive control modalities arises. This paper presents a foot gesture approach centered on robot control constraints to switch between four operating modalities. This control scheme is based on raw data acquired by an instrumented insole located at a human’s foot. It is composed of an inertial measurement unit (IMU) and four force sensors. Firstly, a gesture dictionary was proposed and, from data acquired, a set of 78 features was computed with a statistical approach, and later reduced to 3 via variance analysis ANOVA. Then, the time series collected data were converted into a 2D image and provided as an input for a 2D convolutional neural network (CNN) for the recognition of foot gestures. Every gesture was assimilated to a predefined cobot operating mode. The offline recognition rate appears to be highly dependent on the features to be considered and their spatial representation in 2D image. We achieve a higher recognition rate for a specific representation of features by sets of triangular and rectangular forms. These results were encouraging in the use of CNN to recognize foot gestures, which then will be associated with a command to control an industrial robot.

## 1. Introduction

The agile demand-driven manufacturing process creates the need to design adaptive production using collaborative robotics labelled as cobot. As the flexibility in the manufacturing process increases with the rapid evolution of technology, the fabrication process increases in complexity, impeding standard robots from operating alone. Therefore, operators are brought to work with collaborative robots (cobot) in the same workspace and share with them production activities or working time [1]. This human–robot collaboration is intended to contribute to flexibility and agility thanks to the combination of human’s cognition and management abilities with the robot’s accuracy, speed, and repetitive work [2]. However, cobot’s acceptance in industry is still weak as it raises the thorny issues of security and communication. Safeea et al. [3] demonstrated that the greatest drawback in the development and acceptance of cobots in industries comes from the reliability and the intuitiveness of the proposed interaction scheme. The current trend in research aims to improve interaction by ensuring a smooth control of the robot in an efficient manner at any time through improving working conditions and reducing work-related diseases such as musculoskeletal disorders (MSD) [1].

Assessing such a problem led to third-hand application. An example of such a case can be seen in an assembling process where the operator needs both of his/her hands to complete the task and, thus, the cobot must be able to intervene according to the operator’s need. It is then used to bring parts, hold components while assembling parts, generate a virtual haptic wall to help assembling [1], add new actions with learning by demonstration [4], etc. In such an application, upper body recognition (face recognition and hand gesture) [3,5,6], and lower body recognition (foot-based gesture recognition [7,8,9]) could be used to select the operating mode and execute some remote-controlled motions. Consequently, there will be an interference between the cobot’s motion and the camera’s field of view A facial expression or gesture, such as moving lips, is limited in the number of different commands and it needs a camera to always be oriented to the operator’s face, which is another limitation of the method. Therefore, foot gesture recognition becomes an interesting solution when using instrumented insole including force sensors and inertial measurement unit (IMU).

This project suggests implementing cobot operating mode selection using the foot in the scheduling of production activities to improve manufacturing flexibility. Therefore, an operator could need, utilizing foot gestures, to communicate actions, to be executed by the cobot, and to select different operating modes such as physical collaboration [10], autonomous action in shared activities [11], remote control motion [12], and learning new tasks [4]. This process allows controlling possible modalities of high-dimensionality cobot with a low-dimensionality wearable device such as a smart armband or smart insole.

For this purpose, we suggest a novel method exploiting time series data collected from an instrumented insole in this study. Using these data, a large set of features are computed and then features are extracted based on a dimensionality reduction technic to select relevant ones. Indeed, the relevant features are transformed into a 2D image for classification processing using 2D-CNN. The main contribution of this work lies in the evaluation of the possible spatial representations of the relevant features used in 2D image. The suggested spatial representations allow for the improvement of foot gesture recognition results. As the number of research works in this field is increasing, the next section reviews the state-of-the-art to contextualize the contribution of our research work.

## 2. Related Work

Firstly, a non-exhaustive definition of the cobot as a third-hand is presented. Then, examples of systems using a robot as a collaborative worker are reported. Thereafter, the use of human gesture as command center is explained. Finally, a brief review of the most different existing methods for gesture recognition is analyzed. In this state-of-the-art, the previous studies on foot gestures-based pressure sensor matrices and classification methods, such as CNN, are particularly covered.

### 2.1. Third-Hand Cobot

The third-hand robot is a process developed by Ewerton et al. [13] in which the robot is considered an assistant, i.e., it acts as a third-hand of a human worker. For example, this assistant, also named collaborative robot (or cobot), can provide the necessary tools for its co-worker (the human) to help him/her to perform its tasks. This collaboration can save the worker’s time and energy so that some researches have been conducted to use industrial robots as a third-hand robot. For instance, a semi-autonomous third-hand robot was developed in [14] to assist the human workers in the assembly of furniture. A KUKA-DLR lightweight Robot arm [15] was used as a worker’s third-hand for welding of work pieces in small batches. In this line of thoughts, Metalimbs [16] developed two additional robotic arms to the user’s body, defined as a fourth hand robot, in order to enhance the user’s functions. Another use of cobots is found in the field of retinal microsurgery where the robot shares a tool’s control with the surgeon [17]. Since these cobots need to communicate with humans, the next section explains how gesture helps to achieve such communication.

### 2.2. Use of Human Gesture as Command Center

New intelligent, intuitive, and user-friendly command methods have emerged in the industry and are usually based on direct or indirect contact with the robot. Direct contact interfaces imply physical interaction strategies which include the kinesthetic interfaces or force feedback, allowing them to feel the position, the movements and the forces exerted by the mechanism, and the tactile interfaces which permit to feel the form, the texture, and the temperature [4]. Indirect ones are systems based on artificial vision [18,19], voice recognition [20], and more recently, interfaces using IMU sensors for human gestures recognition [4]. This type of interface is starting to spread in the field of human–robot interaction because it turns out to be more robust to environmental disturbances and constraints such as noise, brightness, etc. [12]. In this study, Neto et al. [12] proposed an interaction strategy based on human gestures captured through IMUs. It permits to recover the specific movements of the upper part of the human body. They offer various modes of interaction depending on whether the human’s posture is static or dynamic. However, such strategies require both hands to be free to operate the robot. Moreover, the results of a comparative study between hand and foot-based gestures in a simulation of a scenario where the hands are busy showed that the use of foot gestures saves more than 70% of time than the traditional approach based on hand gestures. The foot gestures were then perceived as more useful and satisfying [21]. As a result, many current systems use foot gestures as an alternative mechanism of interaction in situations where the hands are preoccupied or unavailable. Some applications use tapping feet and kick to interact with a mobile device [22]. Others use foot-based interaction to produce music [23] or perform navigational tasks in interactive 3D environments [8]. Metalimbs propose an interactive system to control the position of two robotic arms by the movement of the user’s foot and the grip of each arm is controlled by the toes [16]. To achieve such performances, various artificial intelligence algorithms are investigated. As depicted in the next section, gesture classification in the field of artificial intelligence is still an important issue.

### 2.3. Gesture Recognition Methods

Human gesture recognition is applied to recognize the useful information of human motion. Statistical modeling, such as discreet Hidden Markov model (HMM), was used as classifier to learn and recognize five gestures performed during the motor hoses assembly [24]. It was also used to teach robots to reproduce gestures by looking at examples [25], to distinguish between finger and hand gesture classes [26], and to recognize hand gestures in order to command a robot companion [27]. However, HMMs need a large amount of training data and therefore their system performance could be limited by the characteristics of the training data [28]. Dynamic time warping (DTW) is a widely used method in human gesture recognition applications (an algorithm used for online time series recognition). It can deal with gesture signals varying in amplitude and resolve ambiguities in the recognition result even for multiclass classification. It is known that the use of DTW with a set of sequential data of hand gestures have good classification rates [29]. However, it is a dynamic method that focuses most on the local motion information and has less consideration for the global features of gesture trajectories. Contrary to the DTW, the convolutional neural network (CNN) is a recognition method that uses static images of gesture trajectories and, thus, omits the local motion information [30].

Each method has distinct advantages and disadvantages. In fact, both static and dynamic recognition methods (CNN and DTW) were used to achieve better recognition accuracy in digit-writing hand gestures’ localization and recognition for Smart TV systems [30]. However, CNN is more efficient than many traditional classification methods [31]. CNN is known for its robustness at low input variations and low pre-treatment rate necessary for their operation [31]. Numerous applications relying on CNN in the classification of human gestures or actions have been recorded and were based on either 1D-CNN [32,33], 2D-CNN [34,35,36], or 3D CNN [37].

Most applications based on camera rely either on 2D-CNN, as it computes a 2D image as input [36], or 3D-CNN to accurately scope the information in the space. For example, 3D-CNNs have been developed for the recognition of human actions from airport surveillance cameras [37]. This model extracts characteristics of spatial and temporal dimensions by performing 3D convolutions, thus capturing the motion information encoded in several images. Furthermore, for foot-based applications, some research works rely either on 1D-CNN or 2D-CNN when using inertial measurement unit sensors. Those relying on 1D-CNN directly scope the time series signal (data) obtained from the sensors to achieve accurate classification as shown in [32,33]. However, the classification performance is still low as the difficulty to efficiently combine all the information received from the different sensors arises [33]. Furthermore, 2D-CNN appears to be more realistic as it focuses on the analysis based on 2D images rendering it slower than 1D-CNN but more accurate and flexible in the analysis of features extracted from IMU [38]. However, it requires defining the set of images received from raw motion sensors data. Many attempts have been recorded. In [34], a 2D-based CNN method for fall detection using body sensors has been investigated by directly scoping raw motion data in a 2D image without feature extraction and achieving high accuracy of 92.3%. Thus, for the proposed method, there is only scope between two possibilities (fall detection or not). In [35], a similar work has been conducted, based on the effective representation of sEMG (Surface electromyography) signals in images by using a sliding window to continuously address all the signals obtained from the input to a grayscale image. However, none of these proposed works demonstrate the impact of a spatial representation of features used to constitute a 2D image on classification results.

Thus, we formulated two hypothesizes which are (1) for foot-based interaction context, a 2D-convolutional neural network seems to be suitable for foot gesture recognition; and (2) the selection of the most important features and their spatial representation in the 2D image greatly impact the recognition process.

By using an instrumented insole and applying a 2D-CNN algorithm, the main contribution of the present study is to develop a new methodology for a foot gesture recognition system to select a cobot operating mode. The instrumented insole was worn by the worker to acquire the foot gestures’ signals. More specifically, we suggest a simple feature extraction technique using data acquired from an inertial measurement unit (IMU) and force sensors, as well as 2D image generation to classify foot gestures. To achieve this goal, we have evaluated our system in different scenarios of gestures, since those can be performed easily to control a robot. The proposed classification algorithm, trained with backpropagation, is then optimized to recognize gestures. Our results showed a new advance in this area, providing interesting directions for future research by highlighting the impact of features extraction and their spatial representation in a 2D image for the recognition process. By enhancing the existing foot recognition methods, our goal is to increase the ease of work of the operator.

## 3. Materials and Methods

**Figure 1:**
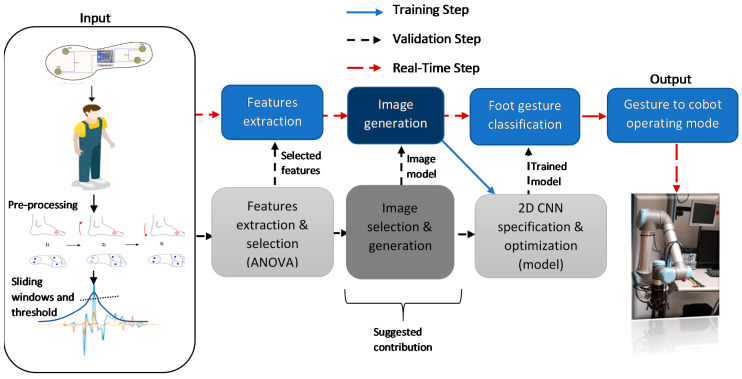
Suggested pipeline for the training, validation, and real-time execution.

Since the operator’s hands were occupied during his work, this article proposes to use foot movements to control a robot. The overview of the proposed gesture recognition system is illustrated in Figure 1.

The system requires data information from a human’s foot to be computed and analyzed for selecting one cobot operating mode. The material aspect is presented in Section 3.1. For the treatment process, the gesture recognition system was based on machine learning classification, thus requiring training and validation phases.

The training phase began with defining a set of foot gestures to be assimilated to cobot operating modes (Section 3.2). Once the dictionary was established, we proceeded to data processing and then features selection (Section 3.3) to reduce the complexity of the model. Once completed, the selected features were transmitted to the image generation (Section 3.3.1) to determine the most relevant representation. The generated images were provided as an input for the 2D-CNN used for foot gestures recognition (Section 3.3.2).

**Figure 2:**
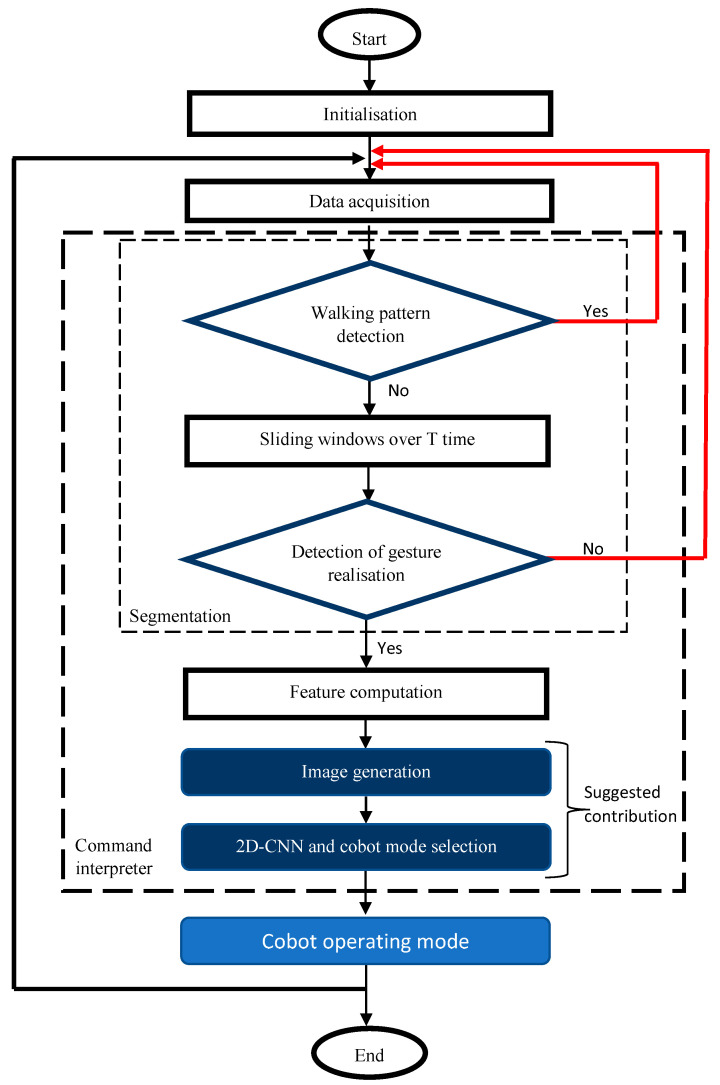
Real-time execution algorithm from data acquisition to cobot operating mode.

The testing phase involved testing the classification of foot gestures with 2D-CNN. The proposed real-time implementation algorithm can be summarized in Figure 2. It depicts an initial set of conditions to discriminate between normal walking pattern and foot gesture command. Once the algorithm detected that the user starts a gesture, it waited for the time T until the gesture was completed. The detection of the start of a gesture was based on a triggering condition related to the FSR’s sensors. Using the data inside the sliding windows, the algorithm proceeded to compute the features, generate an image, perform the 2D CNN classification for gesture recognition, and submit an operating mode to the cobot. The cobot selected an appropriate algorithm from the available operating modes such as trajectory tracking, collision avoidance, etc.

### 3.1. Instrumented Insole

**Figure 3:**
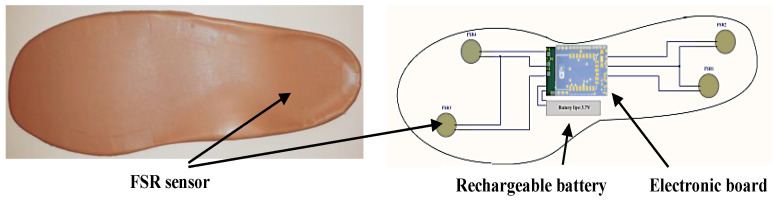
Suggested prototype of the instrumented insole.

While the user made a gesture, the instrumented insole acquired, processed, and wirelessly transmitted the data via TCP to the computer to start the gesture recognition. The proposed enactive insole is a non-intrusive, non-invasive, and inexpensive device. The sampling frequency used in data processing and transmission was 32 Hz (Figure 3). It contained a 9-axis motion processing unit MPU9250 [39], which measured the foot’s acceleration, velocity, and orientation through a set of 3-axis accelerometer, 3-axis gyroscope, and 3-axis magnetometer combined with a digital motion processor (DMP). Moreover, four force-sensitive resistors (FSR), two in the forefoot position and two in heel position, were also integrated to measure the pressure applied on the insole. The analog signals acquired from pressure sensors were converted by an analog-to-digital converter (ADC) ADS1115 [40] with a 16-bit resolution. Finally, an ESP8266-12E WiFi module [41], located at the foot arch position, was used to transmit the data to a local computer. The detailed design of the insole was previously presented in [42].

Once the material architecture was defined, a cobot operating mode based on gesture dictionaries for 2D CNN training phase was used, as presented in the next section.

### 3.2. Foot-Based Command: Gesture Dictionnaries

Selection between cobot operating mode was based on two gesture dictionaries: pressure and IMU sensors (one for each kind of sensor) for classification purposes. Machine learning classification needs a training phase with a set of grayscale images generated by relevant features for each gesture. This section proposes a two-foot-based dictionaries utilizing information from the 3-axis accelerometer, 3-axis gyroscope (angular velocity), 3-axis magnetometer, and the four pressure sensors of the insole.

Based on the sensor readings and different movements of the foot, dictionaries of movements are shown below. Table 1 and Table 2 present some basics movements recognizable by each sensor considered alone.

From these simple foot gestures dictionaries, combinations of three or four movements were used to create five gestures, as shown in Table 3. Each movement has multiple advantages. It was simple to execute and easy to detect at once.

In Table 3, (a) represents an illustration of the first gesture denote (G1) which looks like crushing a cigarette with the forefoot; (b) is an illustration of the second gesture (G2) which looks like crushing a cigarette with the heel; (c) is an illustration of the third gesture (G3) which looks like tap with the forefoot; (d) is an illustration of the fourth gesture (G4) which looks like tap with the heel; and (e) is an illustration of the fifth gesture (G5) which looks like a kick.

Once identified, the foot gestures needed to be mapped with the defined cobot operating mode. In this study, based on observation of Alexander et al. [8], the following commands with mapping gestures are presented in Table 4. Additional gestures with different commands could be certainly defined, as described in the introduction such as physical collaboration [10], autonomous action in shared activities [11], remote control motion [12], and learning new tasks [4].

The proposed foot-based dictionary mapped with cobot operating mode must be decoded in order to accurately scope the difference between gestures. The next section proposes the overall process for data acquisition and features selection.

### 3.3. Data Acquisition and Features Selection

The data presented in Table 3 are acquired by an instrumented insole worn in the left foot. In this study, the gestures of a single participant (one of the authors of this paper, a healthy adult) were recorded. The measurement time of each gesture was set at 15 s. For numerical simulation, signals from the 3-axis accelerometer, 3-axis gyroscope, and the 4 FSRs were exploited. We also measured the Euler angles and the quaternions from the Digital Motion Processor (DMP). The details from the insole’s signals are provided in Table 5.

For this study, we only focused on the sum of FSR sensors rather than considering them alone because, based on our proposed gestures, it is difficult to only have one FSR sensor activated at once.

Once the insole’s data were collected, features enhancement and selection or reduction could be conducted to accurately scope the characteristics of each proposed gesture for classification purposes, thus limiting the complexity of the model [43].

We tried two methods using the proposed dataset. Firstly, we selected 08 features, presented in Table 6, from the acquired data.

The choice of the 08 proposed features was based on our observation of signals behavior for each gesture. We noticed a difference in the signal’s variation for each gesture. Table 7 presents the latter.

At this point, we could observe that the norm of acceleration for G1 and G2 presents important peaks of about 8000 mm·s^−2^. However, for G3 and G4, the value of the peak is lower and equals to 4900 mm·s^−2^. As for G5, the norm of amplitudes attends a higher value for a long time. Moreover, the signals obtained from the norm of the angular velocity, the sum of the two FSR sensors located at the forefoot (*F*1), the sum of the two FSR sensors located at the heel (*F*2), and the Euler Angles are suitable to be selected as different features to discriminate foot gestures.

The second method used in this paper considered feature enhancement and reduction, which consists of using the raw signals obtained from the instrumented insole, and then computed feature enhancement. This operation led to a set of 78 features presented in Table 8 and Table 9.

Dimension reduction technics used in this paper extract the relevant features to be used in the image generation process. According to the state-of-the-art, there are mainly two approaches. One is based on the reduction in features by searching possible combinations of features to identify the principal components with the highest variance which will be used for classification purposes. Usually, the employed method is based on principal component analysis (PCA) which only focuses on generating new inputs, regardless of the label of data, thus posing the problem of the features selection in real-time identification where the principal components might differ from one time to another. The other solution is to deal with features selection which consists of choosing between the set of possible features, the most representatives ones. The method is usually based on statistical analysis in which the evaluation of features importance for discriminating between gestures is realized. In this work, ANOVA statistical analysis, which is the most used in statistical computation, was used to compare the significant differences in characteristics to determine whether or not a characteristic allows good features identification of gestures as suggested in [44]. ANOVA’s result was then calculated from the null hypothesis. The null hypothesis is that all the calculated characteristics distribution is similar. Given that there is a null hypothesis if the probability (*p*-value) is less than 0.05, the characteristics were significantly different. The ANOVA’s results computed with Matlab 2016b for a data set of 100 samples as 20 per gestures are given in Table 10.

**Figure 4:**
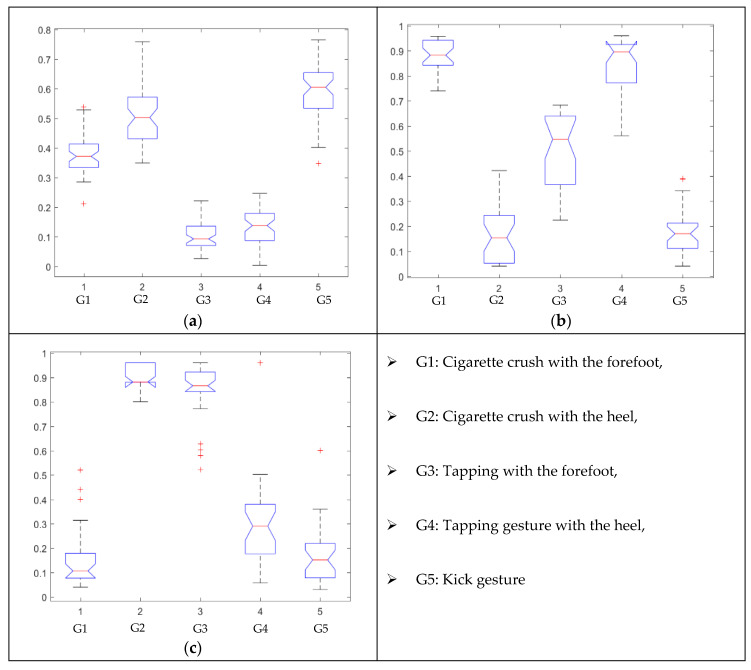
Analysis of variance (ANOVA): (**a**) Mean of the acceleration (*N_am_*), (**b**) Mean of the FSR sensors of the forefoot (*F*1*_m_*), (**c**) Mean of the FSR sensors of the heel (*F*2*_m_*).

For each gesture, ANOVA results determined that there are three main characteristics which are the norm of acceleration (*N_am_*), the sum of the two sensors located at the forefoot (*F*1*_m_*), and the sum of the two FSR sensors located at the heel (*F*2*_m_*). Figure 4 presents the ANOVA representation of each selected feature and its corresponding values for each of the proposed five gestures numerated from G1 to G5.

An analysis of the proposed ANOVA results shows the possibility to enhance our classification method by means of a threshold. Figure 4a shows that, for the mean of the norm of acceleration *Na_m_*, there is a threshold of 0.25. This means that, for gestures where the variation of *Na_m_* is important, such as for gestures 1, 2, and 5, the measured value is greater than 0.25, whereas, for gestures 3 and 4, the value of the *Na_m_* is less than 0.25. Therefore, additional conditions were set.

By reproducing the same analysis, a similar set of conditions applying on the mean of the sum of the two FSR sensors in the heel shows a threshold value of 0.4, meanwhile, for the sum of the two FSR sensors located at the forefoot, the threshold appears to be difficult to be set. A further histogram analysis conducted in a more complete data set of about 100 samples per gesture is presented in Table 11 and Table 12.

Histogram analysis of *Na_m_* shows the same threshold value of 0.25 as the one presented from ANOVA’s result in Figure 4a. The histogram analysis of *F*2*_m_* presents a threshold value of about 0.35 and for *F*1*_m_* the threshold values appear to be 0.38. Those results are mainly the same obtained from ANOVA’s analysis in Figure 4b for *F*1*_m_* and Figure 4c for *F*2*_m_*. In order to generalize the threshold results, we decided to set it to 0.4 for both *F*1*_m_* and *F*2*_m_*. Table 13 presents a summary of the proposed threshold values for the processing algorithm to ensure images normalization.

Once the features are selected, the next section proposes the 2D-CNN image generation for classification purposes.

#### 3.3.1. 2D-CNN Image Generation

A 2D-CNN system was used to recognize gestures. The 2D-CNN system has as an input of a 2D image constituted by the features presented above. Independently from the features selected for image generation, the algorithm of the temporal method involving the signal preprocessing and the image composition follows five steps: (1) collection of the sensor data; (2) segmentation of the signals (the beginning of each gesture was identified and then the first twenty-five pieces of data were recorded from the beginning); (3) determination of all the maximum values of the insole’s sensor measurements; (4) normalization of the data between 0 and 1 (a division of the data by the previously measured maximum); and (5) composition of the matrices of the pixels.

**Figure 5:**
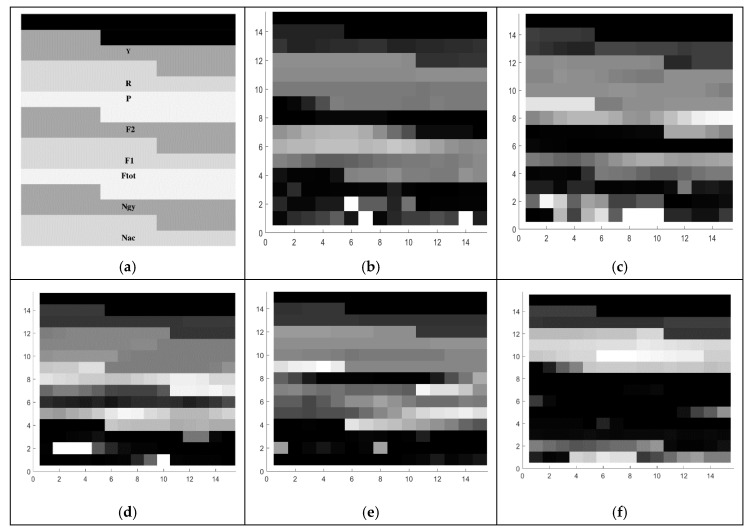
(**a**) Characteristic images of gestures, (**b**) G1: Cigarette crash with the forefoot, (**c**) G2: Cigarette crash with the heel, (**d**) G3: Tapping with the forefoot, (**e**) G4: Tapping gesture with the heel, (**f**) G5: kick gesture.

For 2D-CNN image generation, we firstly define a set of images based on features selection presented in Table 6. These 8 features were represented in an image according to the spatial disposition presented in Figure 5a. This representation results in a 15 × 15 pixels image and the images obtained from the 5 different foot gestures are shown in Figure 5b–f.

**Figure 6:**
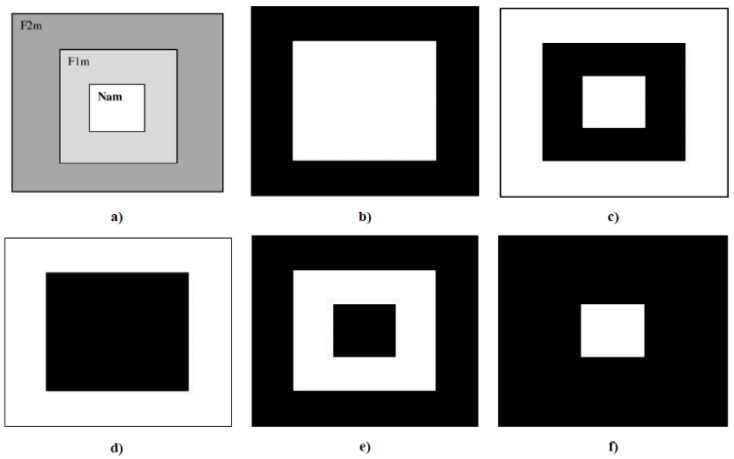
(**a**) Characteristic images of gestures, (**b**) G1: Cigarette crash with the forefoot, (**c**) G2: Cigarette crash with the heel, (**d**) G3: Tapping with the forefoot, (**e**) G4: Tapping gesture with the heel, (**f**) G5: kick gesture.

Secondly, for complexity reduction purposes, we constructed two sets of images based on the three selected features obtained from ANOVA analysis. A first set of images was constructed based on rectangles representation of the selected features according to Figure 6. Each feature was converted into a pixel and displaced accordingly to the representation in Figure 6a. Since they are grayscale images, the value of each pixel in the matrix is between 0 (indicating black) and 255 (indicating white). The images presented in Figure 6 are based on a set of rectangles. Images are also made up of 11 × 11 pixels.

**Figure 7:**
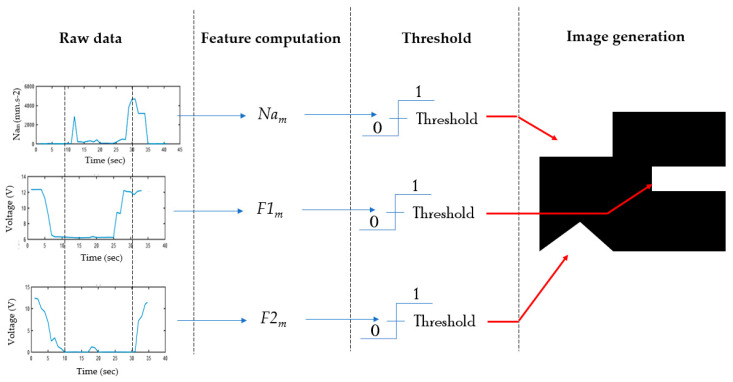
Images generation from selected features.

To reduce the grid size of the image, a new set of geometric representations was proposed for modeling the three selected characteristics. The square, the rectangle, and the triangle represent the mean of the norm of acceleration *Na_m_*, the mean of the sum of two FSR sensors integrated into the forefoot position *F*1*_m_*, and of the two ones integrated in the heel position *F*2*_m_*, respectively. This method is called “Data Wrangling” and it consists of transforming the raw data to another format in order to make it easier to use. Figure 7 presents the proposed method to obtain a set of images to be used. The threshold is determined from the analysis previously presented in Section 3.3.

**Figure 8:**
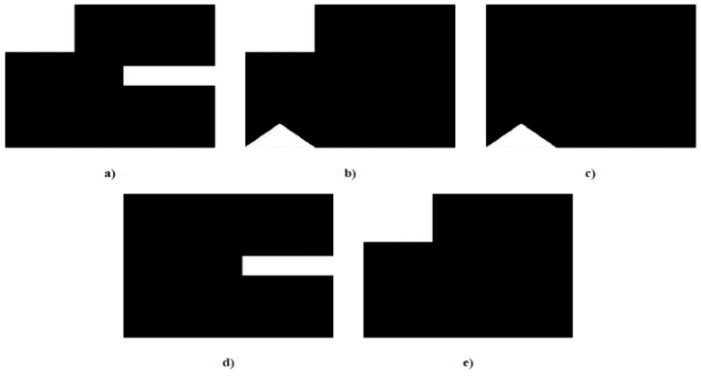
Characteristic images of gestures: (**a**) G1: Cigarette crash with the forefoot; (**b**) G2: Cigarette crash with the heel; (**c**) G3: Tapping with the forefoot; (**d**) G4: Tapping gesture with the heel; (**e**) G5: Kick gesture.

The output of such image generation is a 9 × 9 pixels images that characterizes each gesture. Figure 8 shows the theoretical image obtained for each gesture.

#### 3.3.2. 2D-CNN Classification Method

**Figure 9:**
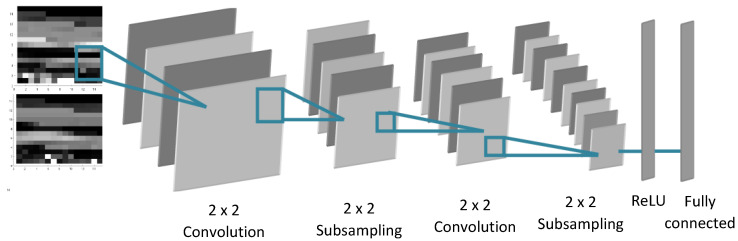
2D-CNN methodology adopted.

A grayscale image was used as an input of CNN. CNN consists of a succession of layers that include feature maps and subsampling maps. The CNN model is designed with four main building blocks, as shown in Figure 9: (1) convolution; (2) pooling or subsampling; (3) non-linearity (ReLU); and (4) fully connected.

Convolution is the first layer of CNN. Indeed, its role consists of extracting the characteristics of the images presented as the input. During this phase, 2D convolution is applied to the image in order to determine its useful information. The filtered images pass through the second layer (pool) of the CNN. The role of this part is to reduce the size of the image while preserving its most important information. Indeed, a sliding window traverses the image and reduces its size by using a local maximum operation. The rectified linear unit (ReLU) is the third layer of the CNN in which each negative value will be replaced by zero. Therefore, the size of the image is not changed in this layer. The fully connected layer is a multilayer perceptron that combines the characteristics of the images and determines the probability of each class presented in the learning phase. In this proposed CNN architecture, the nonlinear function used is the sigmoid function. Figure 9 presents the general structure of the CNN used for gesture recognition.

Based on the structure of image presented as input, there are some characteristics adopted as given in Table 14.

## 4. Results

Foot gesture identification task was considered as a pattern recognition problem in which a set of foot’s movements of one of this paper’s authors was recorded for training and validation steps. The classification of gestures was based on statistical information extracted from its patterns. For every gesture, 70% of data were defined as training samples, 15% as validation samples, and 15% as test ones. The CNN model was trained using the training and validation set and tested independently with the testing set. Many of tests (100) were finally performed to obtain an optimized model. The selected parameters to test the CNN model in TensorFlow were obtained from the training process and were presented for each type of image presented as input. Table 15 presents the latter.

The recognition process is based on the gradient method. Confusion matrix related to each method and the recognition rate for the five-foot gestures are presented in Table 16.

Based on these results, it can be inferred that ANOVA analysis contributed to the great increase (about 14%) in the recognition rate, implying that features specification has an important place in the recognition process. Furthermore, by using the spatial distribution of the selected features obtained from the ANOVA analysis, we achieved different results, 74% for the first case and 100% for the second one. These results show that the rescaling method of the features data has an important impact on the classification base 2D-CNN method.

## 5. Limit of the Study

Limitations in this study can be seen in several points. Firstly, the recognition process only accounts for one user (the first author of this research works) whose characteristic has previously been scoped in the convolutional neural network, thus requiring for every new user to compute the training process. Secondly, our study is conducted in a strictly supervised environment where noises arisen from environmental consideration, such as vibrations, are taken out, thus requiring the enhancement of disturbances robustness for all industries purposes. Thirdly, this current study has not been yet implemented in real-time embedded system for online classification tests. Finally, a study of the proposed classification algorithm for a larger set of gestures and participants is yet to be considered.

## 6. Conclusions and Future Works

In this paper, a new method that can be used for human–robot interaction in hybrid work cells is proposed. The goal is to switch between possible cobot operating modes based on foot gesture command. Therefore, this article presents a foot gesture human–robot interface using an instrumented insole located inside the worker’s left shoe. Firstly, two foot gesture dictionaries were formulated, then five gestures assimilated to five selected commands to control a robot were chosen. Foot gesture signals were collected from the insole and processed for features selection. In this process, a statistical analysis utilizing a dataset recorded from one person who repeated the different foot gestures several times was computed to identify the most representative features, i.e., the mean of the acceleration norm, the mean of the sum of the two FSR sensors located in the forefoot, and the mean of the sum of the two FSR sensors located in the heel. Then several sets of grayscale images based on the spatial representation (geometric form) of the above features in the selected 2D image were proposed to adequately scope the differences between the suggested five gestures. Thus, the proposed 2D images were given as input to a 2D convolutional neural network with backpropagation algorithm for foot gesture recognition. Offline results showed the great impact of variance analysis in the recognition process as we achieve a higher recognition rate of 74% only by selecting the relevant features. Furthermore, a spatial representation of the selected features in the 2D images seems to greatly impact the recognition process as there a set of geometric configurations exists in which the recognition rate is very high, nearly 100%. From these results, it can then be inferred that the use of foot gesture classification for cobot operating mode selection is possible.

Future research aims to increase the number of chosen gestures in order to have more assimilated commands. Furthermore, for globalization purposes, larger sets of foot gesture executions methods from different persons are required and, finally, a real-time implementation of the proposed solution in the instrumented insole processors ought to be attempted.

## Figures and Tables

**Table 1:** Dictionary of detectable movements by the accelerometer with ankle as center.

Movements of Rotation and Translation with Ankle at Center			Movements of Rotation and Translation with Toes at Center
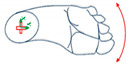 Horizontal movement rotation (with heel as center)	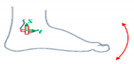 Vertical movement rotation (with ankle as center)	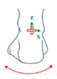 Vertical movement rotation (with ankle as center)	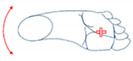 Horizontal movement of rotation (with toes as center)
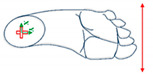 Movement of translation (left/right)	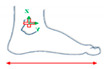 Movement of translation (front/back)	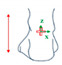 Movement of translation (up/down)	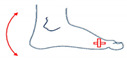 Vertical movement of rotation (with toes as center)

Note: Each movement is described below the illustration.

**Table 2:** Dictionary of captured movement by the pressure sensors.

Active or Inactive Force Sensors (FSR) during the Movements			
4 FSRs	2 FSRs		1 FSR
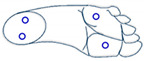 The four sensors are inactive (foot is not touching the ground)	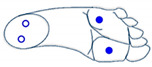 The two sensors at the front are active (foot is inclined forward)	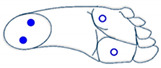 The two sensors at the back are active (foot is inclined backward)	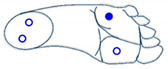 Only the sensor at the front outside is active (foot is inclined front-outward)
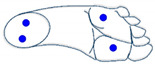 The four sensors are active (foot flat on the ground)	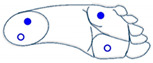 The two outside sensors are active (foot is inclined outwards)	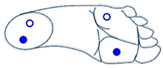 The two inside sensors are active (foot is inclined inwards)	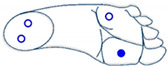 Only the sensor at the front inside is active (foot is inclined front-inward)

Notes: The movements are described below the illustrations. In each illustration (signal sent by pressure sensors), the empty blue circle represents inactive sensor while the full blue circle represents active sensor.

**Table 3:** Representation of the five proposed gestures denoted from G1 to G5.

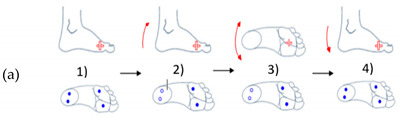	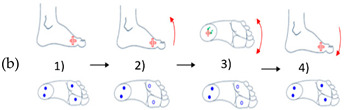
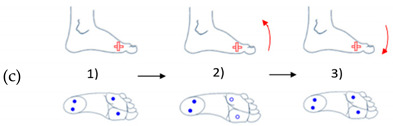	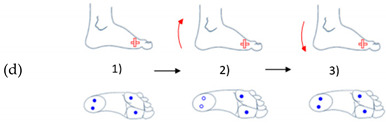
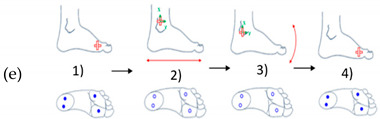	

**Table 4:** Foot mapping gesture.

Foot Gesture	Cobot Operating Mode
1. Cigarette crush with the forefoot	Switching to the “third hand” mode
2. Cigarette crush with the heel	Fast trajectory control
3. Tap with the forefoot	Precise trajectory control (Slow)
4. Tap with the heel	Motor-holding by the robot
5. Kick	Stopping the robot

**Table 5:** Insole’s device signals.

Signal’s Name	Description	Signal’s Origin
*AcX*, *AcY*, *AcZ*	Acceleration in the 03 axis (X, Y, Z)	3-axis accelerometer
*VaX*, *VaY*, *VaZ*	Angular velocity in the 03 axis (X, Y, Z)	3 axis gyroscopes
*P*	Euler’s angle: P (Pitch)	DMP (Digital Motion Processor)
*R*	Euler’s angle: R (Roll)	
*Y*	Euler’s angle: Y (Yaw)	
*q1*, *q2*, *q3*, *q4*	Quaternions	
*F*1	Sum of two FSR sensors located at the forefoot	FSR sensors
*F*2	Sum of two FSR sensors located in the heel	
*Ftot*	Sum of the four FSR sensors	

**Table 6:** Proposed features for foot recognition based on human observation.

Feature’s Name	Description	Signal’s Origin
*Na_m_*	Norm of acceleration	3-axis accelerometer
*Ngy*	Norm of angular velocity	3 axis gyroscopes
*P*	Euler’s angle: P (Pitch)	DMP (Digital Motion Processor)
*R*	Euler’s angle: R (Roll)	
*Y*	Euler’s angle: Y (Yaw)	
*F*1	Sum of two FSR sensors located at the forefoot	FSR sensors
*F*2	Sum of two FSR sensors located in the heel	
*Ftot*	Sum of the four FSR sensors	

**Table 7:** Signals of the norm of acceleration and the norm of angular velocity related to the five proposed gestures.

Gestures	Norm of Acceleration *Na_m_*	Norm of Angular Velocity *Ngy*
G1	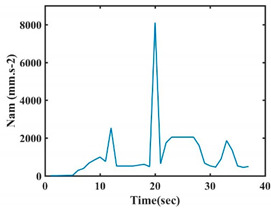	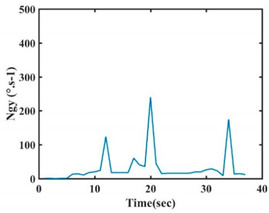
G2	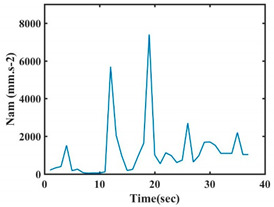	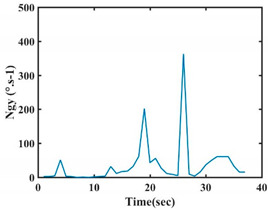
G3	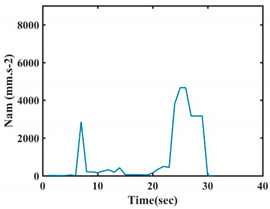	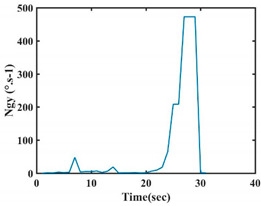
G4	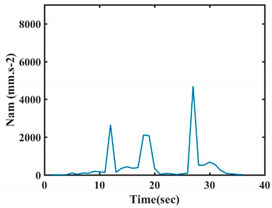	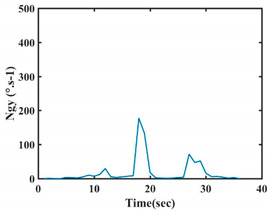
G5	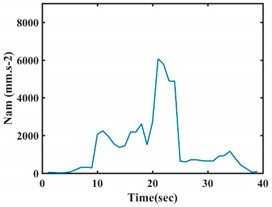	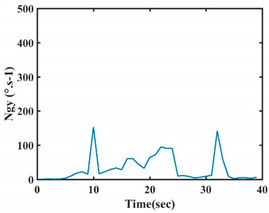

**Table 8:** Features preselected for statistical analysis part 1.

Statistical Parameters (Abbreviation)	Mean (m)	Variance (var)	Standard Deviation (td)
Characteristics	*AcX_m_*, *AcY_m_*, *AcZ_m_*, *N_am_*	*AcX_var_*, *AcY_var_*, *AcZ_var_*	*AcX_std_*, *AcY_std_*, *AcZ_std_*
	*VaX_m_*, *VaY_m_*, *VaZ_m_*	*VaX_var_*, *VaY_var_*, *VaZ_var_*	*VaX_std_*, *VaY_std_*, *VaZ_std_*
	*P_m_*, *R_m_*, *Y_m_*	*P_var_*, *R_var_*, *Y_var_*	*P_std_*, *R_std_*, *Y_std_*
	*q1_m_*, *q2_m_*, *q3_m_*, *q4_m_*	*q1_var_*, *q2_var_*, *q3_var_*, *q4_var_*	*q1_std_*, *q2_std_*, *q3_std_*, *q4_std_*
	*F*1*_m_*, *F*2*_m_*	*F*1*_var_*, *F*2*_var_*	*F*1*_std_*, *F*2*_std_*

**Table 9:** Features preselected for statistical analysis part 2.

Statistical Parameters (Abbreviation)	Skewness (Skew)	Kurtosis (Kurt)	Root Mean Square (Rms)
Characteristics	*AcX_skew_*, *AcY_skew_*, *AcZ_skew_*	*AcX_kurt_*, *AcY_kurt_*, *AcZ_kurt_*	*AcX_rms_*, *AcY_rms_*, *AcZ_rms_*
	*VaX_skew_*, *VaY_skew_*, *VaZ_skew_*	*VaX_kurt_*, *VaY_kurt_*, *VaZ_kurt_*	*VaX_rms_*, *VaY_rms_*, *VaZ_rms_*
	*P_skew_*, *R_skew_*, *Y_skew_*	*P_kurt_*, *R_kurt_*, *Y_kurt_*	*P_rms_*, *R_rms_*, *Y_rms_*
	*q1_skew_*, *q2_skew_*, *q3_skew_*, *q4_skew_*	*q1_kurt_*, *q2_kurt_*, *q3_kurt_*, *q4_kurt_*	*q1_rms_*, *q2 _rms_*, *q3 _rms_*, *q4 _rms_*
	*F*1*_skew_*, *F*2*_skew_*	*F*1*_kurt_*, *F*2*_kurt_*	*F*1*_rms_*, *F*2*_rms_*

Notes: *Ac* and *Va* correspond, respectively, to the acceleration and the angular velocity computed along the X, Y, Z axis; *Na* is the norm of the acceleration; *P*, *R*, and *Y* are the Euler angles; *q1*, *q2*, *q3*, *q4* are the Quaternions.

**Table 10:** ANOVA’s statistical results.

Characteristics	ANOVA (*p*-Value)	Characteristics	ANOVA (*p*-Value)	Characteristics	ANOVA (*p*-Value)
*AcX_m_*	0.0362	*AcX_var_*	9.82089 × 10^−9^	*AcX_std_*	1.48053 × 10^−12^
*AcY_m_*	0.0037	*AcY_var_*	7.0831 × 10^−13^	*AcY_std_*	8.60749 × 10^−14^
*AcZ_m_*	0.1522	*AcZ_var_*	1.11004 × 10^−13^	*AcZ_std_*	7.66846 × 10^−15^
*VaX_m_*	0.7163	*VaX_var_*	0.0006	*VaX_std_*	0.0004
*VaY_m_*	4.2743 × 10^−5^	*VaY_var_*	0.0078	*VaY_std_*	0.0001
*VaZ_m_*	0.6465	*VaZ_var_*	0.9967	*VaZ_std_*	3.58713 × 10^−6^
*P_m_*	4.70768 × 10^−17^	*P_var_*	0.0005	*P_std_*	1.15232 × 10^−9^
*R_m_*	1.99492 × 10^−35^	*R_var_*	2.12177 × 10^−16^	*R_std_*	1.01984 × 10^−5^
*Y_m_*	0.0006	*Y_var_*	0.0008	*Y_std_*	2.06642 × 10^−9^
*q1_m_*	1.44179 × 10^−16^	*q1_var_*	6.40077 × 10^−11^	*q1_std_*	2.37864 × 10^−6^
*q2_m_*	2.29963 × 10^−19^	*q2_var_*	1.97067 × 10^−10^	*q2_std_*	2.38143 × 10^−12^
*q3_m_*	1.78075 × 10^−9^	*q3_var_*	0.048	*q3_std_*	9.1542 × 10^−13^
*q4_m_*	1.19374 × 10^−7^	*q4_var_*	7.41653 × 10^−7^	*q4_std_*	1.13254 × 10^−10^
*F*1*_m_*	7.62104 × 10^−67^	*F*1*_var_*	1.52173 × 10^−16^	*F*1*_std_*	1.56796 × 10^−12^
*F*2*_m_*	1.64653 × 10^−65^	*F*2*_var_*	3.33183 × 10^−17^	*F*2*_std_*	3.23638 × 10^−8^
*AcX_rms_*	0.0104	*AcX_kurt_*	1.54661 × 10^−9^	*AcX_skew_*	1.48053 × 10^−12^
*AcY_rms_*	0.0024	*AcY_kurt_*	4.18682 × 10^−21^	*AcY_skew_*	8.60749 × 10^−14^
*AcZ_rms_*	0.1614	*AcZ_kurt_*	2.44817 × 10^−17^	*AcZ_skew_*	7.66846 × 10^−15^
*VaX_rms_*	0.5866	*VaX_kurt_*	8.66958 × 10^−7^	*VaX_skew_*	0.0004
*VaY_rms_*	2.09045 × 10^−5^	*VaY_kurt_*	7.08042 × 10^−14^	*VaY_skew_*	0.0001
*VaZ_rms_*	0.6497	*VaZ_kurt_*	3.37356 × 10^−6^	*VaZ_skew_*	3.58713 × 10^−6^
*P_rms_*	1.48031 × 10^−13^	*P_kurt_*	0.0671	*P_skew_*	1.15232 × 10^−9^
*R_rms_*	0.0618	*R_kurt_*	0.5788	*R_skew_*	1.01984 × 10^−5^
*Y_rms_*	0.0003	*Y_kurt_*	0.1283	*Y_skew_*	2.06642 × 10^−9^
*q1_rms_*	3.92505 × 10^−13^	*q1_kurt_*	0.0284	*q1_skew_*	2.37864 × 10^−6^
*q2 _rms_*	0.1313	*q2* *_kurt_*	0.6328	*q2* *_skew_*	2.38143 × 10^−12^
*q3 _rms_*	0.091	*q3* *_kurt_*	0.0146	*q3* *_skew_*	9.1542 × 10^−13^
*q4 _rms_*	6.92425 × 10^−11^	*q4* *_kurt_*	0.0152	*q4* *_skew_*	1.13254 × 10^−10^
*F*1*_rms_*	6.59022 × 10^−49^	*F*1*_kurt_*	1.66156 × 10^−7^	*F*1*_skew_*	1.56796 × 10^−12^
*F*2*_rms_*	2.68932 × 10^−38^	*F*2*_kurt_*	5.70291 × 10^−5^	*F*2*_skew_*	3.23638 × 10^−8^
*N_am_*	1.49825 × 10^−115^				

**Table 11:** Histogram analysis of *Na_m_* and *F*2*_m_*.

	*Na_m_*	*F*2*_m_*
G1	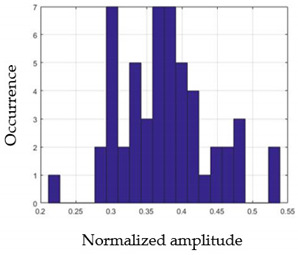	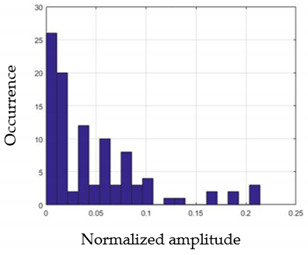
G2	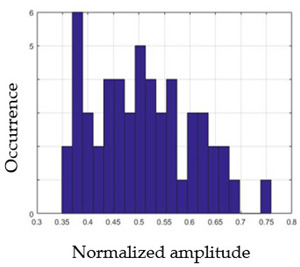	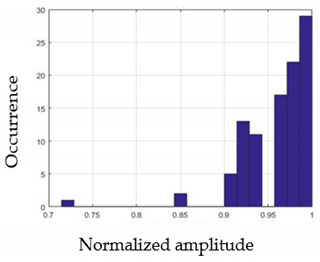
G3	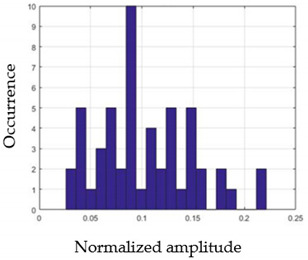	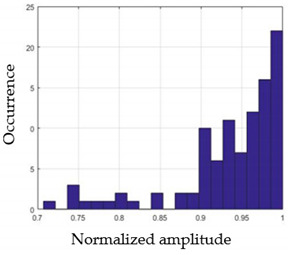
G4	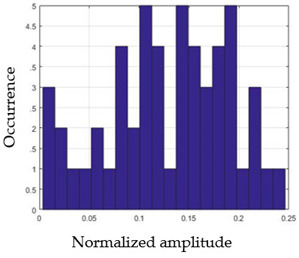	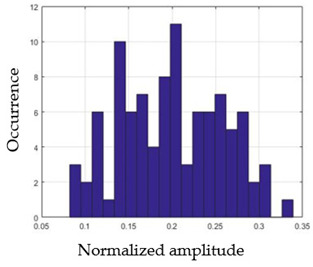
G5	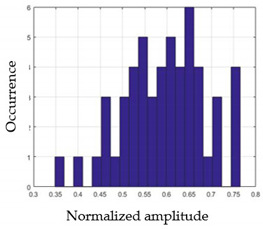	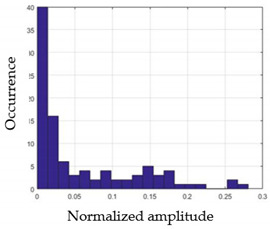

**Table 12:** Histogram analysis of *F*1*_m_*.

G1	G2
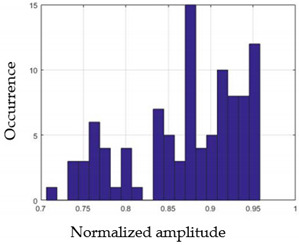	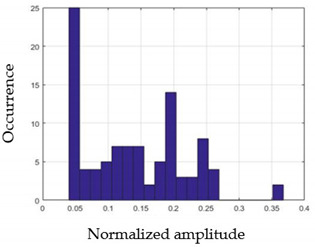
G3	G4
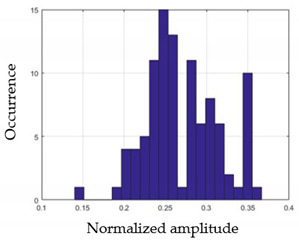	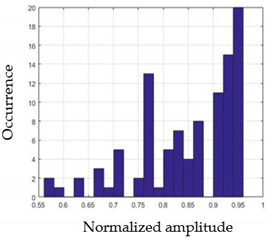
G5	
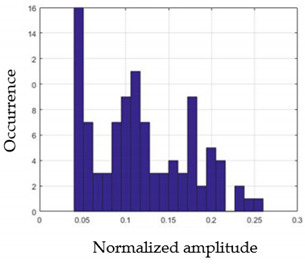	

**Table 13:** Selected features threshold.

Mean of the acceleration norm (*Na_m_*)	$\{\begin{array}{c} \mathrm{If}\;(N_{am}>0.25)\;\mathrm{then}\;N_{am}=0.9\\ \mathrm{If}\;\mathrm{not}\;(N_{am}<0.25)\;\mathrm{then}\;N_{am}=0.1 \end{array}$
Mean of the sum of two FSR sensors in the forefoot (*F*1*_m_*)	$\{\begin{array}{c} \mathrm{If}\;(F1_{m}>0.4)\;\mathrm{then}\;F1_{m}=0.9\\ \mathrm{If}\;\mathrm{not}\;(F1_{m}<0.4)\;\mathrm{then}\;F1_{m}=0.1 \end{array}$
Mean of the sum of two FSR sensors at the heel (*F*2*_m_*)	$\{\begin{array}{c} \mathrm{If}\;(F2_{m}>0.4)\;\mathrm{then}\;F2_{m}=0.9\\ \mathrm{If}\;\mathrm{not}\;(F2_{m}<0.4)\;\mathrm{then}\;F2_{m}=0.1 \end{array}$

**Table 14:** CNN characteristics.

		Convolution C1	Pooling P1	Convolution C2	Pooling P2
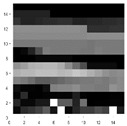 (10 neurons on the Fully connected Layer)	Number of convolution Kernel	5	/	15	/
	Windows size	2 × 2	2 × 2	2 × 2	2 × 2
	Input	15 × 15	14 × 14	7 × 7	6 × 6
	Output	14 × 14	7 × 7	6 × 6	3 × 3
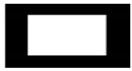 (11 neurons on the Fully connected Layer)	Number of convolution Kernel	7	/	17	/
	Windows size	2 × 2	2 × 2	2 × 2	2 × 2
	Input	11 × 11	10 × 10	5 × 5	4 × 4
	Output	10 × 10	5 × 5	4 × 4	2 × 2
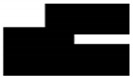 (100 neurons on the Fully connected Layer)	Number of convolution Kernel	10	/	10	/
	Windows size	4 × 4	2 × 2	2 × 2	2 × 2
	Input	9 × 9	6 × 6	3 × 3	2 × 2
	Output	6 × 6	3 × 3	2 × 2	1 × 1

**Table 15:** CNN test parameters.

**Parameters**	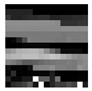	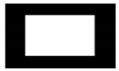	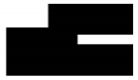
**Learning Rate**	0.01	0.00019	0.005
**Momentum Coefficient**	0.6	0.899	0.9

**Table 16:** 2D-CNN classification results.

Images Input	Recognition Rate	Comments
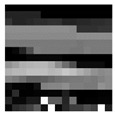 Image based on temporal analysis	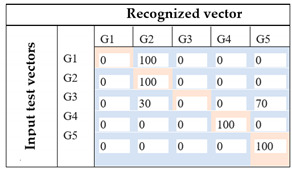	The recognition rate is about 60%. By exploiting all the 8 features from human observation, only 3 foot gestures are correctly recognized (G2, G4, G5). For gesture recognition, G1 and G2 are recognized as the same gesture. Furthermore, the system could not accurately identify G3 because there is 30% of cases where G3 is classified as G2 and 70% where G3 is classified as G5.
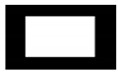 Image based on ANOVA features First attempt: (Set of rectangles)	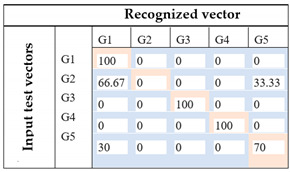	By using statistical analysis based on ANOVA, the recognition rate appears to be greater than the previous one for about 14%. This set of images based on a spatial representation of selected features using a rectangular form could successfully recognize 3 foot gestures (G1, G3, G4). Furthermore, the system is able to make a clear distinction between G1 and G2. However, there is still some confusion of G2, between G1 and G5, and G5, between G1 and G5, with 66.6%, 33.3%, 30%, and 70%, respectively.
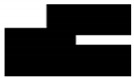 Image based on ANOVA features Final proposition: (Set of rectangles and triangles)	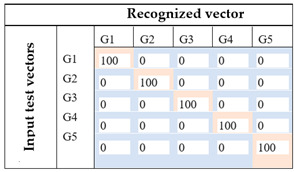	With the enhancement of the images using ANOVA for selecting feature and the modification of the spatial representation of the features in the images using a set of forms (squares, rectangles, and triangles), the system achieves a 100% of recognition rate. Therefore, each foot gesture is correctly identified.

## Data Availability

Data is available in the Supplementary File.

## Supplementary Materials

Supplementary Materials are available online at https://www.mdpi.com/article/10.3390/s21175743/s1.
